# Early Insights From a Digitally Enhanced Diabetes Self-Management Education and Support Program: Single-Arm Nonrandomized Trial

**DOI:** 10.2196/25295

**Published:** 2021-02-22

**Authors:** Folasade Wilson-Anumudu, Ryan Quan, Cynthia Castro Sweet, Christian Cerrada, Jessie Juusola, Michael Turken, Carolyn Bradner Jasik

**Affiliations:** 1 Omada Health, Inc San Francisco, CA United States; 2 Evidation Health, Inc San Mateo, CA United States

**Keywords:** diabetes education, digital health, remote monitoring, type 2 diabetes

## Abstract

**Background:**

Translation of diabetes self-management education and support (DSMES) into a digital format can improve access, but few digital programs have demonstrated outcomes using rigorous evaluation metrics.

**Objective:**

The aim of this study was to evaluate the impact of a digital DSMES program on hemoglobin A_1c_ (HbA_1c_) for people with type 2 diabetes.

**Methods:**

A single-arm, nonrandomized trial was performed to evaluate a digital DSMES program that includes remote monitoring and lifestyle change, in addition to comprehensive diabetes education staffed by a diabetes specialist. A sample of 195 participants were recruited using an online research platform (Achievement Studies, Evidation Health Inc). The primary outcome was change in laboratory-tested HbA_1c_ from baseline to 4 months, and secondary outcomes included change in lipids, diabetes distress, and medication adherence.

**Results:**

At baseline, participants had a mean HbA_1c_ of 8.9% (SD 1.9) and mean BMI of 37.5 kg/m^2^ (SD 8.3). The average age was 45.1 years (SD 8.9), 70% were women, and 67% were White. At 4-month follow up, the HbA_1c_ decreased by 0.8% (*P*<.001, 95% CI –1.1 to –0.5) for the total population and decreased by 1.4% (*P*<.001, 95% CI –1.8 to –0.9) for those with an HbA_1c_ of >9.0% at baseline. Diabetes distress and medication adherence were also significantly improved between baseline and follow up.

**Conclusions:**

This study provides early evidence that a digitally enhanced DSMES program improves HbA_1c_ and disease self-management outcomes.

## Introduction

### Background

Over 34 million people in the United States have diabetes (9% of the adult population), and 1 in 4 health care dollars spent in the United States is for diabetes care [1]. Among all diabetes cases, 90%-95% are type 2 diabetes mellitus (T2DM) [2]. A core component of diabetes management is comprehensive diabetes self-management education and support (DSMES), which is associated with improved outcomes and lower costs [3-5]. DSMES is traditionally delivered in person, either one on one or in a group setting with a certified diabetes care and education specialist (CDCES).

DSMES is widely covered by private and public insurance, including Medicare, and is typically prescribed by a physician at diagnosis, when education gaps exist, or when the treatment plan is changed. The primary goal of DSMES is to help patients acquire the knowledge, skills, and abilities for diabetes self-care [6]. Core educational topics include disease awareness, glucose monitoring, medication adherence, nutrition support, delay of complications, and problem-solving [7].

Despite the widely accepted benefits of DSMES, access remains a challenge. Only 43 states and 57% of counties in those states have accredited DSMES programs in the United States [8]. As of 2017, only 52% of people diagnosed with diabetes in the United States have accessed self-management support services, with rates decreasing in recent years [9]. To address the unmet need, technology-enabled platforms have emerged as a more accessible venue for DSMES delivery. There are numerous commercial products available that allow people to access DSMES programs through personal mobile devices (eg, smartphones, tablets, laptops) with a wide range of approaches [10,11]. Staffing varies widely from none (100% patient-driven) to uncredentialed coaches to CDCES.

Technology-based DSMES programs have demonstrated a positive impact on hemoglobin A_1c_ (HbA_1c_) in academic settings with noncommercially available programs [12]. These interventions typically adhere to DSMES guidelines and include credentialed staff for program delivery. Commercially available technology-based DSMES solutions in the market are often limited by lack of accreditation, uncredentialed staff, and research results produced from less rigorous methods [13]. Although some studies have demonstrated that commercially available DSMES programs improve diabetes-related outcomes for users, the staffing, number of touchpoints, manner of delivery (asynchronous vs synchronous), and inclusion of connected devices, among other factors, vary widely among programs [14-16]. As such, more research is needed to understand best practices for digital DSMES delivery. Furthermore, methodologically rigorous research is also needed to demonstrate the parity of outcomes to in-person care [12].

### Objective

The goal of this pilot study was to evaluate the impact of a digital DSMES program enhanced with deep lifestyle and behavior change support on HbA_1c_ for people with T2DM and elevated HbA_1c_. We hypothesized that the digital DSMES program would be associated with greater improvements in HbA_1c_ for people who were furthest away from their HbA_1c_ goal (baseline HbA_1c_≥9.0%) at the start of the program. We further evaluated the impact of the digital DSMES program on cardiovascular and patient-reported outcomes, as cardiovascular risk factors are a frequent comorbidity of diabetes.

## Methods

### Participants

We invited members of an online health community to participate in this study (Achievement, Evidation Health Inc). Achievement is a web- and mobile-based community in the United States where members can connect their activity trackers, and fitness and health apps to the platform and, by logging activities, accumulate points that are redeemable for monetary rewards. Additionally, members self-report on various health conditions and are invited to participate in remote research opportunities as relevant studies become available. In this study, recruitment was targeted to members who had self-reported a diagnosis of T2DM. Invited members were linked to an online research study platform (Achievement Studies, Evidation Health Inc) where study eligibility was assessed using automated screener questions. Individuals who lived in the United States, were at least 18 years of age, self-reported a T2DM diagnosis, self-reported HbA_1c_ of 7.5% or greater, had a BMI≥25 kg/m^2^ (≥23 kg/m^2^ if they self-identified as Asian), and had access to a computer or smartphone to participate in the digital DSMES program were eligible for the study.

### Procedures

If deemed eligible after completing the screener, potential participants continued in the online study platform to sign an electronic informed consent form and completed an online baseline survey, which consisted of questions about their demographics, health and diabetes history, and patient-reported outcomes. They then completed a baseline visit at a Quest Diagnostics Patient Service Center (PSC) of their choosing. The baseline visit consisted of a venous whole blood draw, physical measurements (height, weight, waist circumference), resting blood pressure, and resting heart rate. After completing the PSC visit, potential participants were instructed to set up their account on the digital DSMES program. After completion of a signed electronic informed consent form, and both the PSC visit and program account setup, individuals were considered enrolled in the study. Participants were able to reach out to research staff with questions via email or phone through the online study platform before and during the enrollment process, and could continue to reach out throughout the study.

During the study period, participants were encouraged to engage with the DSMES program. All participants were provided a cellularly connected weight scale that was linked to their program account. Participants who were advised to use monitoring devices in their diabetes self-care were provided cellularly connected blood pressure monitors and glucose meters. Participants were also able to access their own personal online study platform dashboard to complete study procedures and keep track of their progress throughout the study through the use of any web-enabled device. Approximately 4 months after enrollment, participants repeated the online survey and clinical outcome measures (HbA_1c_, blood pressure). Participants received compensation for completing each study-related task such as surveys and lab visits. This study was approved by the Western Institutional Review Board (Puyallup, WA).

### Study Outcomes

The primary outcome of this study was change in HbA_1c_ from baseline to 4 months, as well as changes in HbA_1c_ based on starting HbA_1c_ values. Secondary outcomes included changes in cardiovascular risk factors (blood pressure, total cholesterol [TC]) among those who started the study with elevated risk factors, in addition to changes in diabetes distress and medication adherence from baseline to 4 months.

### Measurements

At baseline, participants completed an assessment at the PSC that included 13 mL venous whole blood specimen collection under sterile conditions by a trained phlebotomist. The nonfasting blood specimens were processed for HbA_1c_ and a lipids panel (TC, high- and low-density lipoprotein [HDL, LDL], and TC/HDL ratio). A trained technician collected blood pressure after a 5-minute quiet resting period with legs uncrossed using an automatic blood pressure monitor and size-adjustable cuff. Height was measured to the nearest centimeter using a calibrated stadiometer with the participant in stocking feet. Weight was measured using a calibrated scale with the participant in light clothing and no shoes. Waist circumference was measured in whole units (inches) using a nonstretchable measuring tape above the first layer of clothing. BMI was calculated from weight in kilograms divided by height in meters squared. Results were sent by Quest Diagnostics and accessed by the research team via secure file transfer. Participants received copies of their results both via secure email and mail.

Participants completed an online survey of patient-reported outcomes including the Diabetes Distress Scale (DDS), a 17-item scale of different dimensions of distress and burden related to diabetes, which has been shown to have reliability and validity [17], and the Simplified Medication Adherence Questionnaire (SMAQ), a 6-item measure that categorizes respondents as adherent or nonadherent based on recent patterns of medication-taking behaviors [18].

The original protocol planned for a repeat assessment using identical methods 4 months after enrollment. However, the 4-month assessments were scheduled to begin in April of 2020, during the height of the COVID-19 pandemic [19]. People with diabetes are at high risk for severe illness from COVID-19 [20]; therefore, the study protocol was changed to eliminate the in-person visit to support participants to shelter in place. In replacement of the venipuncture blood draw, a Quest Diagnostics Qcard self-collection card was sent to each participant for collection of HbA_1c_ and blood lipids data. The Qcard is a self-collection card that uses the dried blood spot method, with a correlation to venipuncture HbA_1c_ in the range of 0.95 to 1.0 [21]. Triglycerides and LDL were not available through the Qcard and as such were removed as study outcomes. Weight at the 4-month time point was collected using a cellularly connected scale (BodyTrace Inc, Palo Alto, CA, USA) that was provided to every participant in the program. Participants who were given home blood pressure monitors (BodyTrace, Inc) in the program were asked to use them to collect the 4-month blood pressure reading. Blood pressure monitors were sent to participants who did not get the devices at the program start and were given instructions for collecting resting blood pressure at home at 4 months. The post-test self-report online survey was identical to the baseline survey.

### Intervention

Omada for Diabetes is a digitally enhanced DSMES program designed to build self-management skills and support diabetes management between outpatient visits with primary care providers and specialists to ensure that users achieve their health targets (eg, HbA_1c_, blood pressure, cholesterol) and obtain health maintenance services (eg, screening for neuropathy and retinopathy). The program offers disease education, comprehensive lifestyle self-management support (ie, support for weight loss, dietary changes, physical activity increases), support for involvement in members’ current medication regimen, and support for use of monitors or trackers for their blood sugar and blood pressure, which are often used to inform small modifications in food intake, physical activity, medication, or communication with health care providers. Participants used a technology-enabled platform with a portable interface to a variety of personal mobile devices. All participants received a cellularly connected BodyTrace weight scale, and if needed, a blood glucose monitor (3G BioTel Care, Telcare LLC, Concord, MA) was also provided. Participants were assigned to a CDCES who provided individualized coaching around the American Association of Diabetes Educators 7 self-care behaviors [22]. They were also placed in a virtual peer group including other program participants with T2DM, and could communicate with peers through a secure discussion board. As needed, the CDCES referred participants back to their primary care team for medication reviews or adjustments as their health targets and self-care goals were achieved. The program is accredited by the Association of Diabetes Care and Education Specialists [23]. The program takes a user-centered approach that encourages participants to engage at a time and frequency they choose, and with the tools and resources they find most useful, and does not have any predetermined volume or pattern that participants are expected to engage in program features.

### Statistical Analysis

The study was powered to detect a clinically meaningful 0.5% reduction in the primary outcome of HbA_1c_. With an estimated standard deviation of 1.8 and power set to 90%, the minimal sample size needed was 162. To allow for potential 20% loss to follow up and 10% of lab HbA_1c_ values being below 7.5% at baseline, a total of 186 participants were planned for enrollment.

Descriptive statistics are presented to describe the demographics and baseline health status of participants. Baseline correlations using Pearson and Spearman correlation coefficients were examined to determine variables (age, gender, BMI) that could potentially confound HbA_1c_ outcomes. No significant correlations were detected; therefore, paired *t* tests were used to examine baseline to post-test differences in study outcomes. Post hoc analyses were performed to examine the change in HbA_1c_ based on the starting HbA_1c_ range, with the hypothesis that those with higher blood glucose levels may receive greater benefit. Elevated blood pressure and blood lipids were not among the criteria for study inclusion and were therefore assessed as secondary outcomes of interest; we examined changes specifically among those who began the study with elevated cardiovascular risk factors. The McNemar test was performed to examine the change in the proportion of the population that was adherent to medications from baseline to post-test. Program engagement is summarized using averages across several metrics to reflect how participants engaged with the program over the course of the 4-month study.

We analyzed outcomes using complete case analysis for those who returned 4-month clinical and patient-reported survey data. Using multiple imputation, with an imputation of baseline values for primary and secondary outcomes for those with missing data at 4 months, we found that outcomes were similar in magnitude and statistical significance using both analytic methods. Therefore, we present our findings on the sample using results from the complete case analysis.

## Results

### Study Recruitment

**Figure 1:**
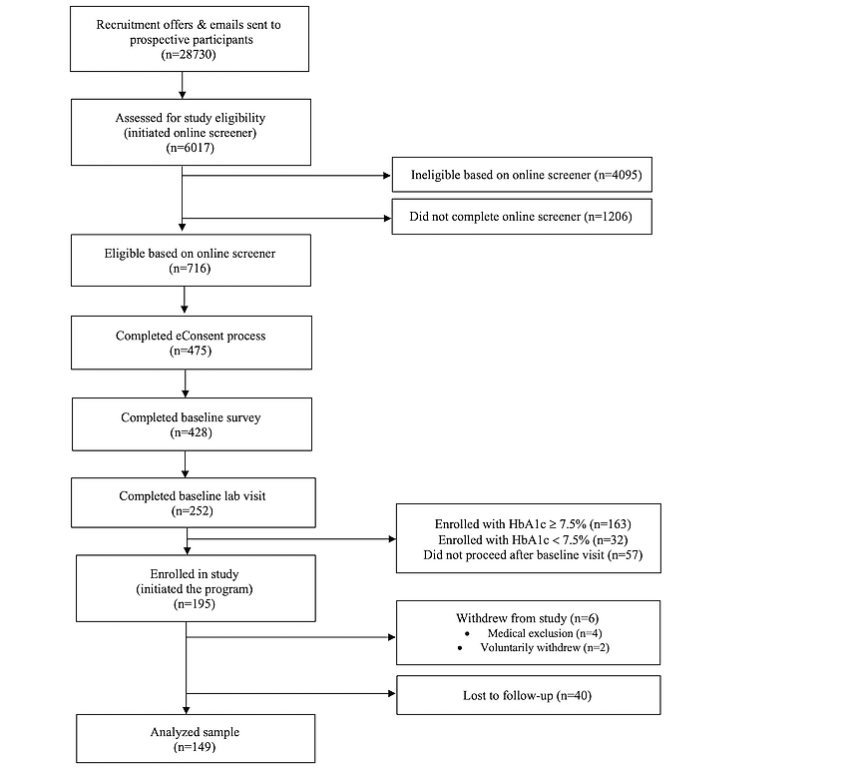
Study participant flowchart. HbA_1c_: hemoglobin A_1c_.

Although the recruitment goal was 162 participants with starting HbA_1c_ above 7.5%, 32 of the first 100 participants’ laboratory HbA_1c_ result was below the 7.5% threshold. Therefore, we changed the protocol to use the baseline HbA_1c_ as a clinical criterion for the study and only accepted those with a lab HbA_1c_ value of 7.5% or greater. We continued enrollment until we reached at least 162 participants with a baseline HbA_1c_ of 7.5% or greater and allowed the 32 participants with a baseline HbA_1c_ below 7.5% to remain in the study. The final enrolled sample was 195, including 163 with a baseline HbA_1c_ of 7.5% or greater and 32 with a baseline HbA_1c_ of less than 7.5%. Six participants were withdrawn from the study: 4 developed a medical condition that precluded participation and 2 requested to voluntarily withdraw. At post-test, 78.8% (n=149) of the remaining 189 participants completed the home test kit; 8 were not sent kits as they resided in states where the home test is not authorized for distribution, and 88.4% (n=167) completed the online questionnaire. Study completion was defined as a final HbA_1c_ value or completion of the final online questionnaire. We compared baseline demographic and clinical values for participants who completed the 4-month data collection and those who were lost to follow up, and found no significant differences across any baseline characteristics. We define loss to follow up as incompletion of the primary outcome of HbA_1c_. See Figure 1 for the flow of participants through each stage of the study.

### Participant Characteristics at Baseline

Baseline characteristics of participants are shown in Table 1. The average starting HbA_1c_ was 8.9%; 50% began the study with an HbA_1c_ of 9.0% or higher. The mean age was 45.1 years, and the majority of participants were female and White. On average, total cholesterol was in the normal range, and blood pressure was close to the nationally recommended goal for those with diabetes. As measured by the SMAQ, 19% of participants were adherent to their current medication regimen. The mean DDS score at baseline was 2.7. A total or subscale score >2.0 (moderate distress) is considered clinically meaningful; average scores <2.0 reflect little or no distress, between 2.0 and 2.9 reflect moderate distress, and ≥3.0 reflect high distress [24].

**Table 1:** Baseline participant characteristics (N=195).

Baseline characteristic^a^		Value
Age (years), mean (SD)		45.1 (8.9)
Female, n (%)		136 (69.7)
**Race/ethnicity, n (%)**		
	White/Caucasian	131 (67.2)
	Black/African American	32 (16.4)
	Hispanic or Latino	17 (8.7)
	Asian	6 (3.1)
	American Indian or Alaska Native	2 (1.0)
	Native Hawaiian or other Pacific Islander	1 (0.5)
	Other	6 (3.1)
BMI, mean (SD)		37.5 (8.3)
Weight (pounds), mean (SD)		235.6 (57.3)
Weight (kg), mean (SD)		106.9 (26.0)
Hemoglobin A_1c_, mean (SD)		8.9 (1.9)
Total cholesterol (mg/dL), mean (SD)		178.9 (43.3)
Systolic blood pressure (mmHg), mean (SD)		127.0 (16.1)
Diastolic blood pressure (mmHg), mean (SD)		82.0 (10.4)
Diabetes Distress Score, mean (SD)		2.7 (1.0)
Adherent to current medications, n (%)		36 (18.5)

^a^There were no statistically significant differences across baseline characteristics among those with and without follow-up data.

### Program Engagement

Averaged across the 16 program weeks, participants used their blood glucose meter an average of 7.4 times per week. Participants weighed in an average of 4.9 times per week, interacted with their CDCES an average of 1.6 times per week, completed an average of 0.8 lessons per week, interacted with their peer groups an average of 0.9 times per week, tracked their physical activity 5.3 times per week, and tracked meals an average of 10.2 times per week.

### Diabetes Outcomes

Baseline to post-test changes in all study outcomes are shown in Table 2. Among all participants who completed both a baseline and 4-month HbA_1c_ test (n=149), participants achieved a statistically significant decrease in HbA_1c_ of 0.8% (*t*_148_ =–6.2, *P*<.001). Table 3 shows changes based on starting HbA_1c_ values. Those who started the study with an HbA_1c_ of 9.0% or higher saw the greatest magnitude of change, with an average decrease of 1.4% (*t*_72_ =–6.1, *P*<.001). Across the total sample, weight significantly decreased an average of 3.0 pounds over 4 months (*t*_146_ =–2.2, *P*=.03), and 18.4% of the sample achieved significant weight loss (>5% body weight) (Table 2).

**Table 2:** Baseline to post-test changes in clinical outcomes (N=167).

Outcomes			n	Baseline	Post-test	Difference	95% CI	*P* value
**Total sample^a^**								
	HbA_1c_^b^ (%)		149	8.9	8.1	–0.8	–1.1 to –0.5	<.001
	Weight (pounds)		147	231.4	228.3	–3.0	–5.8 to –0.3	.03
	Weight (kg)		147	105.0	103.6	–1.4	–2.6 to –0.1	.03
	5% weight loss (%)		147	0.0	18.4	18.4	0.1 to 0.2	<.001
	**Diabetes Distress Scale**		167	2.6	2.3	–0.3	–0.5 to –0.2	<.001
		Emotional Burden	167	2.7	2.4	–0.3	–0.5 to –0.1	<.001
		Physician-Related	167	2.1	1.8	–0.3	–0.4 to –0.1	.001
		Regimen-Related	167	3.0	2.6	–0.4	–0.6 to –0.3	<.001
		Interpersonal	167	2.7	2.4	–0.3	–0.5 to –0.1	.002
	Medication adherence (%)		158	20.3	31.0	10.7	—^c^	.01
**Elevated risk subsample^d^**								
	TC^e^ (mg/dL)		43	230.0	190.5	–39.5	–51.3 to –27.6	<.001
	SBP^f^ (mmHg)		114	131.6	132.5	0.9	–2.1 to 3.9	.54
	DBP^g^ (mmHg)		114	84.7	82.0	–2.7	–4.3 to –1.0	.002

^a^Study participants with complete data from both baseline and 4-month time points.

^b^HbA_1c_: hemoglobin A_1c_.

^c^—:Not applicable.

^d^Study participants who began the study with elevated cardiovascular risk factors.

^e^TC: total cholesterol.

^f^SBP: systolic blood pressure.

^g^DBP: diastolic blood pressure.

**Table 3:** Baseline to post-test changes in hemoglobin A_1c_ (HbA_1c_) based on starting HbA_1c_.

HbA_1c_ category	n	Baseline	Post-test	Difference	95% CI	*P* value
<7.5%	24	6.3	6.4	0.1	–0.2 to 0.4	.49
7.5%-7.9%	24	7.7	7.4	–0.3	–0.6 to 0.1	.18
8.0%-8.9%	28	8.4	7.8	–0.6	–1.0 to –0.2	.002
>9.0%	73	10.4	9.0	–1.4	–1.8 to –0.9	<.001

### Cardiovascular Outcomes

At baseline, 58.5% (114/195) of the participants had systolic or diastolic blood pressure above the normal range (<120 mmHg and <80 mmHg, respectively). There was no significant change in systolic blood pressure, whereas diastolic blood pressure decreased by an average of 2.7 mmHg (*t*_113_=–3.2, *P*=.002). Only 43 participants had elevated TC above 200 mg/dL at baseline, and a significant decrease was found post-test (*t*_42_=–6.7, *P*<.001) (Table 2).

### Patient-Reported Outcomes

In the total sample, diabetes distress significantly decreased from 2.6 at baseline to 2.3 at post-test (*t*_166_=4.5, *P*<.001; Table 2). Significant improvements in distress were observed across all DDS subscales (*P<.*01). The proportion of the sample adherent to their medication regimen increased from 20% at baseline to 31% at post-test (McNemar *χ*^2^_1,158_=7.0, *P*=.01).

## Discussion

### Principal Findings

The results of this study provide initial evidence that the enhanced digital DSMES program was effective for improving HbAlc, weight, diabetes distress, and medication adherence among a sample of people with T2DM and elevated HbA_1c_. Furthermore, those who were furthest from their HbA_1c_ goal at the start of the program (baseline HbA_1c_≥9.0%) achieved the greatest improvement in HbA_1c_, with an average change of 1.4%.

We found an inconsistent impact on cardiovascular outcomes among participants who started the study with elevated risk factors, with some improvements in diastolic blood pressure and TC, but no improvements in systolic blood pressure. However, blood pressure at baseline was close to the nationally recommended goal for those with diabetes, and the program was not designed to address hypertension specifically. Engagement was strong as evidenced by the high frequency of use across the features of the digital platform.

These results are consistent with prior studies of digital DSMES programs (both academic and commercial) that showed improvements in HbA_1c_ and psychosocial outcomes [3,25-28]. In particular, the magnitude of the HbA_1c_ reduction in this program is comparable to that of prior studies. Kumar et al [15] reported an HbA_1c_ reduction of 0.86% and a higher effect in those with a higher baseline HbA_1c_. Dixon et al [16] reported a higher reduction in HbA_1c_ by baseline group, but the intervention also included medication titration and physician support. This study adds to the growing evidence that digital DSMES significantly improves HbA_1c_, and can also impact weight loss and cholesterol [12,29].

The clinical outcomes observed in this study meet or exceed those expected from traditional DSMES programs as set by the American Diabetes Association [30], as well as more resource-intensive digitally delivered programs that combine DSMES with physician telehealth services [16]. Further, the high rates of participant engagement with the program highlight many of the benefits of continuously accessible DSMES.

The improvements in medication adherence are encouraging given that this is a major challenge in diabetes management [31-33]. Digital delivery offers unique opportunities for patient engagement around improving medication-taking behaviors, as CDCES staff can be more proactive and support medication use in a timelier manner. Mobile apps can surface more frequent screenings, follow up, and in-app tracking to identify issues sooner so that a CDCES can reach out and provide education and support.

### Limitations

There were several limitations to this pilot study. First, this pilot study is limited by its single-arm design and therefore carries the typical challenges in a nonrandomized design of unknown causal inference. Future research will benefit from a control group comparison and a randomized design to allow for a maximally rigorous test of the intervention. Second, we had to change the study methodology for follow-up lab measurement due to COVID-19 by shifting to a self-collected blood specimen versus a phlebotomist-collected venipuncture specimen; this creates potential for measurement error between instruments. However, this risk is attenuated by the high correlation of the venipuncture HbA_1c_ and dried blood spot method [21]. Third, it is possible that the study sample recruited may not be fully representative or generalizable of the population of people living with diabetes, as participants self-selected from the online health community into the research opportunity. However, the clinical criteria (ie, HbA_1c_ outside of the desired therapeutic range) increases the likelihood that study participants were individuals who would benefit from better diabetes self-management. Despite the high rates of program engagement observed among participants across the 4-month study, expectations around engagement in digital health studies remain exploratory, with varying definitions of meaningful engagement across digital platforms.

### Conclusions

This study provides additional evidence that a digitally delivered DSMES program enhanced with deep lifestyle and behavior change support impacts HbA_1c_ for people with T2DM and elevated HbA_1c_, showing the greatest benefit for those with higher blood glucose levels, and suggests benefits for weight loss and improvements in cardiovascular outcomes. Future research is needed to understand the potential impact of digital DSMES on long-term diabetes outcomes to meet the needs of the changing health care landscape.

## Abbreviations

CDCES: certified diabetes care and education specialist
DDS: Diabetes Distress Scale
DSMES: diabetes self-management education and support
HbA_1c_: hemoglobin A_1c_
HDL: high-density lipoprotein
LDL: low-density lipoprotein
PSC: Patient Service Center
SMAQ: Simplified Medication Adherence Questionnaire
T2DM: type 2 diabetes mellitus
TC: total cholesterol
